# Long-Acting Cabotegravir and Rilpivirine in Patients With HIV With Solid Organ Transplantation: A Case Series

**DOI:** 10.1093/ofid/ofaf470

**Published:** 2025-08-18

**Authors:** Ana Moreno, Ruben Fernandez-Ibanez, Santos Del Campo, Maria J Perez-Elias, Jose L Casado, Miguel Garcia, Manuel Velez, Maria J Vivancos, Santiago Moreno

**Affiliations:** Department of Infectious Diseases, University Hospital Ramón y Cajal, IRYCIS, Madrid, Spain; CIBERINFEC, Instituto de Salud Carlos III, Madrid, Spain; Department of Infectious Diseases, University Hospital Ramón y Cajal, IRYCIS, Madrid, Spain; Department of Infectious Diseases, University Hospital Ramón y Cajal, IRYCIS, Madrid, Spain; Department of Infectious Diseases, University Hospital Ramón y Cajal, IRYCIS, Madrid, Spain; CIBERINFEC, Instituto de Salud Carlos III, Madrid, Spain; Department of Infectious Diseases, University Hospital Ramón y Cajal, IRYCIS, Madrid, Spain; CIBERINFEC, Instituto de Salud Carlos III, Madrid, Spain; Gastroenterology Department (Liver Transplant Unit), University Hospital Ramon y Cajal, Madrid, Spain; Pharmacy Department, University Hospital Ramon y Cajal, Madrid, Spain; Department of Infectious Diseases, University Hospital Ramón y Cajal, IRYCIS, Madrid, Spain; CIBERINFEC, Instituto de Salud Carlos III, Madrid, Spain; Department of Infectious Diseases, University Hospital Ramón y Cajal, IRYCIS, Madrid, Spain; CIBERINFEC, Instituto de Salud Carlos III, Madrid, Spain; Gastroenterology Department (Liver Transplant Unit), University Hospital Ramon y Cajal, Madrid, Spain

**Keywords:** cabotegravir, HIV, long-acting drugs, rilpivirine, solid organ transplantation

## Abstract

**Background:**

Managing individuals with both HIV infection and a history of solid organ transplantation presents unique challenges due to interactions between antiretroviral therapy and immunosuppressive regimens. Long-acting injectable therapies may offer advantages in reducing drug interactions and improving adherence.

**Methods:**

This retrospective study assessed the virological efficacy and safety of long-acting injectable therapy with a combination of 2 antiviral agents in 5 patients with stable HIV infection who had undergone kidney or liver transplantation. Patients were followed for up to 74 weeks after initiating therapy. Virological response, immunological parameters, renal and hepatic function, and immunosuppressive drug levels were monitored.

**Results:**

All patients maintained undetectable viral loads throughout the study period, with no virological failure or drug-resistance development. CD4^+^ T-cell counts remained stable, and no clinically significant changes in renal or hepatic function were observed. Immunosuppressive drug levels remained within the therapeutic range without requiring dose adjustments. No patient experienced severe adverse effects or injection-site complications, and adherence was high throughout the study.

**Conclusions:**

Long-acting injectable therapy was effective and well tolerated in individuals with stable HIV infection following solid organ transplantation. The findings suggest that this approach may be a viable treatment option, reducing drug interactions while maintaining virological suppression. Further research with larger cohorts is needed to confirm these findings and establish guidelines for implementation in transplant recipients.

## Teaser

The study evaluates the virological efficacy and safety of long-acting cabotegravir and rilpivirine (CAB + RPV LA) in HIV-infected solid organ transplant recipients. Findings suggest sustained viral suppression, stable immunosuppressive drug levels, and no significant drug interactions, supporting its potential as a therapeutic option.

Patients living with HIV who undergo solid organ transplantation (SOT) face complex management challenges due to potential interactions between antiretroviral therapy (ART) and immunosuppressive agents. The introduction of integrase strand transfer inhibitors (INSTIs) has helped reduce these interactions, which were more frequent with older ART such as non-nucleoside reverse transcriptase inhibitors (NNRTIs) and especially boosted protease inhibitors [1]. Despite major advances since the first transplant cohort in 2003, maintaining virological control, adequate immunosuppression, and preventing opportunistic infections remain difficult.

These patients often present with comorbidities that further complicate management. Chronic kidney disease (CKD) and liver failure are particularly important due to their impact on drug metabolism [2]. These conditions have traditionally been linked to reduced immunosuppressive exposure, increased mortality, shortened graft survival, and a higher risk of acute rejection [2, 3]. However, more recent data suggest that with appropriate patient selection and modern ART, outcomes have improved substantially, with patient and graft survival now approaching those of HIV-negative recipients [4].

Managing multiple medications increases the risk of drug–drug interactions. Many immunosuppressive agents are metabolized by cytochrome P450 enzymes [5], which can be affected by ART. Current guidelines recommend selecting regimens with low-interaction potential, favoring oral second-generation INSTIs, such as bictegravir or dolutegravir, and avoiding protease inhibitors or NNRTI-based regimens [6, 7].

Long-acting injectable cabotegravir and rilpivirine (CAB + RPV LA), introduced in 2021 [8], may offer an alternative in this setting. Although guidelines advise against using CAB + RPV LA as a bridging strategy when oral intake is temporarily compromised posttransplant, this recommendation refers to short-term surgical contexts rather than its long-term role in maintaining viral suppression [6, 9].

CAB + RPV LA could help simplify treatment by reducing pill burden and minimizing intestinal and first-pass metabolism interactions. These agents have demonstrated good safety and efficacy in people living with HIV outside complex clinical settings [10], but their role in transplant recipients remains largely unexplored. A single published case report has described the use of CAB + RPV LA in a patient with HIV undergoing intermittent hemodialysis who later received a kidney transplant [11]. However, this was a correspondence describing 1 patient without structured follow-up. To our knowledge, no other cases have been reported, and this is the first case series evaluating the use of long-acting ART in solid organ transplant recipients living with HIV. We describe 5 such cases and discuss key considerations for their clinical management.

## METHODS

A descriptive analysis has been conducted to evaluate the virological efficacy and safety of the CAB + RPV LA combination as a therapeutic option in patients with HIV infection and SOT. This retrospective study included 5 patients treated at the HIV outpatient clinic of Ramón y Cajal University Hospital, Madrid, Spain, between September 2023 and January 2025.

CAB + RPV LA was administered via an initial intramuscular loading dose, followed by injections every 8 weeks, without an oral initiation phase. Inclusion criteria considered patients with liver or kidney transplantation, prior stable virological suppression (HIV RNA <50 copies/mL), and no evidence of previous failure with NNRTIs. Patients with comorbidities or clinical conditions contraindicating the use of CAB + RPV LA were excluded.

Relevant demographic and clinical data were collected, including age, sex, time from transplant to initiation of CAB + RPV LA, comorbidities, immunosuppressive therapies, and previous antiretroviral treatments. Virological efficacy was assessed through HIV viral load (HIV RNA) and CD4^+^ lymphocyte count. Safety was analyzed by recording adverse events (AEs) related to CAB + RPV LA and their impact on continued treatment.

Clinical follow-up was performed over a period of up to 74 weeks from the initiation of therapy. The primary outcomes evaluated were the maintenance of virological suppression (HIV RNA <50 copies/mL), the presence of viral rebounds, variations in CD4+ count, body mass index (BMI), glomerular filtration rate (GFR), and the incidence of local or systemic AEs.

This study adhered to current ethical guidelines and ensured the confidentiality of patients' personal data, in accordance with the General Data Protection Regulation and the directives of the Hospital's Ethics Committee for Research with Medicinal Products. Since this was a retrospective analysis in which data were handled in a fully anonymized manner, informed consent from patients was not required. This approach was approved by the ethics committee, ensuring that the study met all applicable legal and ethical requirements.

## RESULTS

### Characteristics of the Patients

A total of 5 patients with SOT and HIV infection were included in this study (Table 1). The cohort comprised 4 liver transplant recipients and 1 kidney transplant recipient. The median age at the time of enrollment was 60 years (range: 49–61), and 4 out of 5 patients were male. All patients were on immunosuppressive therapy, including everolimus, cyclosporine, tacrolimus, or mycophenolate mofetil. The median time from transplantation to initiation of CAB + RPV LA was 6 years (range: 1–12).

**Table 1.** Baseline Characteristics of Patients Prior to Treatment With Long-Acting Cabotegravir and Rilpivirine

	Patient 1	Patient 2	Patient 3	Patient 4	Patient 5
Type of transplant	Liver	Liver	Liver	Liver	Renal
Age (y)	60	61	49	59	61
Sex	Male	Female	Female	Male	Male
Race	White	White	White	White	Gypsy
HIV risk factor	IDU	IDU	IDU	IDU	IDU
CDC stage	C3	C3	B3	C3	B3
Baseline BMI	22	21	38	25	32
CD4 T-cell count (cells/mL)	856	575	767	347	583
HIV RNA (log_10_ copies/mL)	<1.30	<1.30	<1.30	<1.30	<1.30
Baseline GFR (mL/min)	76	32	91	85	24
Years on ART	24	28	14	29	28
Number of prior ARV lines	7	6	8	11	22
Prior NNRTI experience	NVP No failure	No	EFV, RPV No failure	NVP, ETRA, RPV No failure	EFV, RPV No failure
Last ARV regimen	BIC/TAF/FTC	BIC/TAF/FTC	DTG/3TC	BIC/TAF/FTC	BIC/TAF/FTC
Reason for LA	Patient’s request	Medical proposal (convenience, remote ART delivery)	Medical proposal (convenience, bimonthly pharmacy visits)	Medical proposal (proposed as an alternative option)	Medical proposal (GFR of 24 mL/min on BIC/TAF/FTC)
Years from trasplantation to LA	5	10	12	6	1
LA start date	23 September 2023	2 November 2023	6 November 2023	9 February 2024	20 May 2024
HBV status	HBsAg− HBcAb+ HBsAb+	HBsAg− HBcAb+ HBsAb+	HBsAg− HBcAb+ HBsAb+	HBsAb+ (vaccinated)	HBsAb+ (vaccinated)
IS regimen and dose (baseline and follow-up)	EVE 0.5 mg-0-0.25 mg	CsA 75 mg-0-75 mg + MMF 500 mg-0-500 mg	TAC 2 mg/alt days with 1.5 mg + MMF 500 mg/d	EVE 0.75 mg/12 h + GC 4 mg/d	CsA 25 mg-0-50 mg + MMF 360 mg/12 h + GC 5 mg/d
Comorbidities	COPD T2DM Dyslipemia	CKD	Depression Obesity HTN Dyslipemia	HPV-related ORL cancer HTN Depression	Cirrhosis T2DM Ischemic cardiomyopathy HTN Dyslipemia
Concomitant therapies	Pantoprazole AAS Salbutamol (inh) Vildagliptin/metformin Atorvastatin Vitamin D	Pantoprazole Enalapril Clonazepam Vitamin D	Atorvastatin Lorazepam Enalapril Pregabalin Vitamin D Semaglutide	Pantoprazole Candesartan Sildenafil AAS Lorazepam Vitamin D	Isosorbide mononitrate Epleronone Verapamile Betahistidine Losartan Ezetimibe Linagliptine AAS

Abbreviations: HIV, human immunodeficiency virus; ART, antiretroviral therapy; LA, long-acting; BMI, body mass index; CD4, cluster of differentiation 4; RNA, ribonucleic acid; GFR, glomerular filtration rate; NNRTI, non-nucleoside reverse transcriptase inhibitors; IS, immunosuppressive therapy; NVP, nevirapine; EFV, efavirenz; RPV, rilpivirine; ETRA, etravirine; ARV, antiretroviral; BIC, bictegravir; TAF, tenofovir alafenamide; FTC, emtricitabine; DTG,: dolutegravir; 3TC, lamivudine; CsA, cyclosporine A; MMF, mycophenolate mofetil; EVE, everolimus; GC, glucocorticoids; HBV, hepatitis B virus; HBsAg, hepatitis B surface antigen; HBcAb, hepatitis B core antibody; HBsAb, hepatitis B surface antibody; HTN, hypertension; ORL, otorhinolaryngologic; T2DM, type 2 diabetes mellitus; COPD, chronic obstructive pulmonary disease; AAS, acetylsalicylic acid; IDU, injection drug use; Inh, inhaler.

Comorbidities were common among the participants, with CKD, hypertension, dyslipidemia, type 2 diabetes mellitus, and depression being the most frequently reported conditions. Baseline BMI varied among participants, ranging from 21 to 38, with a median BMI of 25. Two individuals met the criteria for obesity at the start of therapy.

Three patients had serologic evidence of past HBV infection. None of them showed an isolated anti-HBc pattern, and HBV DNA testing was not performed, as there were no clinical indications or risk factors suggesting occult hepatitis B. It is worth noting that active HBV infection represents a contraindication for CAB + RPV LA due to the risk of viral reactivation, but none of the patients met this criterion.

All patients had long-standing HIV infection with a median duration of 28 years (range: 14–29) on ART. HIV subtype was unknown for all the patients. All of them had also previously been treated with multiple ART regimens, with a median of 8 prior regimens (range: 6–22), including INSTIs and NNRTIs. Baseline CD4^+^ T-cell count ranged from 347 to 856 cells/mm^3^, with all patients maintaining suppressed HIV RNA levels below 50 copies/mL.

### Virological and Immunological Efficacy

**Figure 1.**
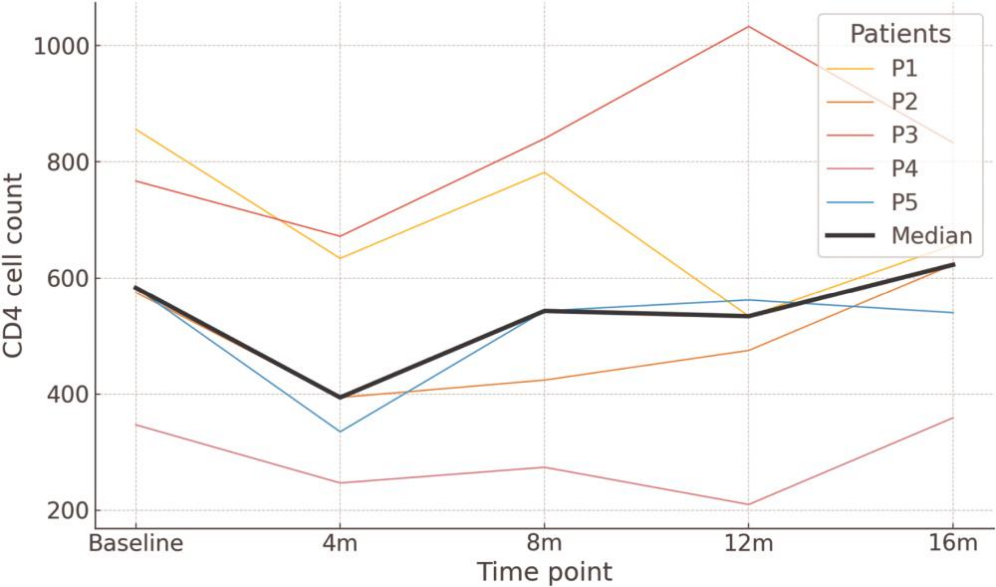
CD4 cell count trajectories over time following switch to CAB + RPV LA. Individual CD4^+^ T-cell counts for each of the 5 patients at baseline, 4, 8, 12, and 16 m following CAB + RPV LA initiation. Thin colored lines represent individual patients (P1–P5), and the thick black line indicates the cohort median at each time point.

CD4^+^ T-cell count trajectories for each individual are shown in Figure 1. Median CD4^+^ count at baseline was 583 cells/mm^3^ (range: 347–856), and values remained stable throughout the follow-up period, with medians of 394, 543, 475, and 540 cells/mm^3^ at 4, 8, 12, and 16 months, respectively. The last recorded CD4^+^ count ranged from 359 to 1033 cells/mm^3^. These fluctuations were not associated with virological failure or clinical deterioration.

All patients maintained virological suppression throughout the follow-up period. HIV RNA levels remained consistently <1.30 log_10_ copies/mL at all time points. Across the cohort, a total of 20 HIV RNA determinations were performed during follow-up, including baseline measurements at the time of CAB + RPV LA initiation. Each patient underwent between 3 and 5 viral load assessments. The median duration of follow-up from initiation to last measurement was 8.9 months (range: 7.9–15.5 months), with a mean monitoring frequency of 1 determination every 2.7 months. All patients were virologically suppressed at baseline, and no blips or rebounds were detected throughout the monitoring period.

### Safety Outcomes

**Figure 2.**
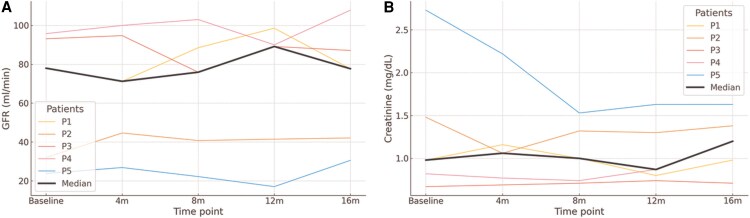
Renal function marker trajectories over time following switch to CAB + RPV LA. (*A*) Glomerular filtration rate. (*B*) Serum creatinine levels. Individual glomerular filtration rate and serum creatinine levels for each of the 5 patients at baseline, 4, 8, 12, and 16 m following CAB + RPV LA initiation. Thin colored lines represent individual patients (P1–P5), and the thick black line indicates the cohort median at each time point.

Renal function, as measured by the estimated GFR and serum creatinine levels, showed stability or slight improvement in most patients (Figure 2*A* and 2*B*). The median baseline estimated GFR was 78.01 mL/min (32.07–93.16), compared with 77.75 mL/min (42.11–87.13) at 16 months. Similarly, serum creatinine levels showed no deterioration, with a median baseline value of 0.98 mg/dL (0.82–1.48) and 0.98 mg/dL (0.87–1.38) at 16 months. All values are expressed as median (interquartile range). No episodes of rejection were observed in any patient during the follow-up period.

**Figure 3.**
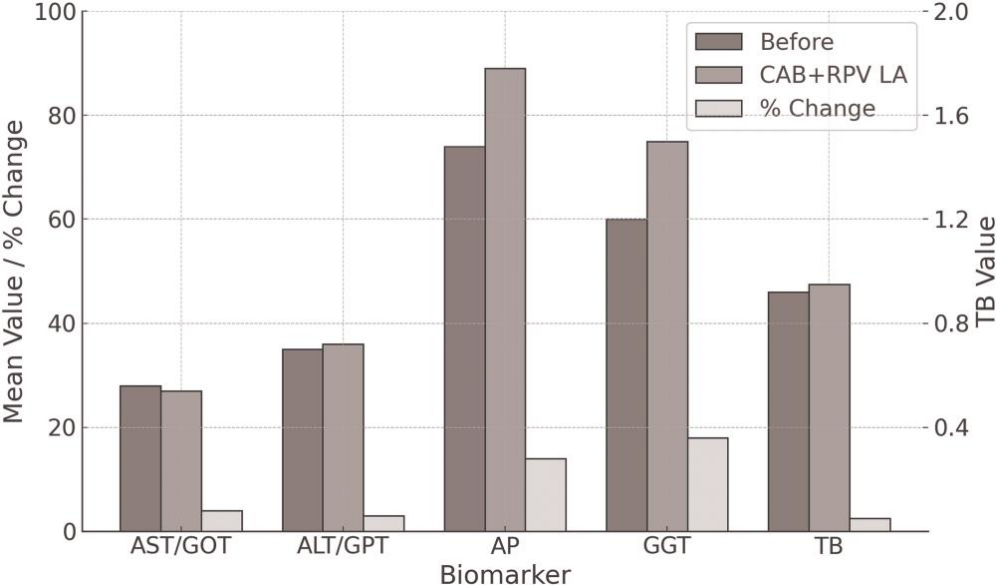
Mean changes in liver function markers for 5 patients with SOT before and during CAB + RPV LA therapy.

Liver function markers (including aspartate aminotransferase [AST], alanine aminotransferase [ALT], alkaline phosphatase [AP], GGT, and total bilirubin) remained within expected limits throughout follow-up. Individual trajectories and percentage changes are shown in Figure 3, with no clinically significant hepatotoxicity observed in any patient.

### Immunosuppressive Drug Concentrations

Therapeutic plasma levels of immunosuppressive drugs remained stable during treatment with CAB + RPV LA (Table 2). Everolimus, cyclosporine, and tacrolimus levels demonstrated minor fluctuations, with median percentage changes of 0.19, 0.06, and 0.04, respectively. These changes did not necessitate dose adjustments or modifications in the immunosuppressive regimen.

**Table 2.** Changes in Immunosuppressive Therapy Levels After CAB + RPV LA Treatment

	P1	P2	P3	P4	P5
Measure	EVE (ng/mL)	CsA (ng/mL)	TAC (ng/mL)	EVE (ng/mL)	CsA (ng/mL)
Median IS therapy CAB + RPV LA (IQR)	5.68 (4.37–6.54)	99.00 (79.98–104.35)	4.60 (3.92–5.33)	4.15 (3.70–5.00)	92.50 (71.70–134.10)
Median IS therapy before (IQR)	4.76 (3.67–5.49)	93.12 (75.23–98.16)	4.41 (3.77–5.11)	4.04 (3.60–4.87)	83.29 (64.56–120.75)
% change in IS therapy	0.19	0.06	0.04	0.03	0.11

Each column corresponds to an individual patient (P1–P5), with their respective immunosuppressive drug listed below. Values represent the intrapatient median (interquartile range, IQR) of plasma drug concentrations during the 12 m prior to switching to CAB + RPV LA, compared with the median (IQR) during follow-up after the switch. The % change reflects the relative difference in median levels before and after CAB + RPV LA initiation. The timing of drug level sampling in relation to CAB + RPV LA injections was not uniformly recorded; thus, the pharmacokinetic context of the measured concentrations remains uncertain.

Abbreviations: IS, immunosuppressive; CAB + RPV LA, clinical/renal laboratory analysis; EVE, everolimus; CsA, cyclosporine A; TAC, tacrolimus.

## DISCUSSION

These results suggest that CAB + RPV LA is effective and safe in HIV-infected solid organ transplant recipients. Despite comorbidities and polypharmacy, the regimen maintained virological suppression, CD4^+^ count, and stable immunosuppressive drug levels. Given the limited experience, further research is needed.

Pharmacological interactions between immunosuppressants, antimicrobials, and ART are critical in this population. None of our patients experienced adverse reactions or significant pharmacokinetic changes requiring intervention, despite the known weak interaction with calcineurin inhibitors [12]. A recent study [13] described interactions between rilpivirine and mycophenolate, sirolimus, and prednisone, mainly via CYP450, with minimal involvement of transporters. Caution remains warranted with other drugs, such as azole antifungals and common antimicrobials, commonly used for prophylaxis or treatment [5, 14].

Although theoretical concerns exist, major drug–drug interactions are unlikely due to LA-ART's minimal involvement in intestinal or first-pass metabolism and weak modulation of relevant enzymatic pathways [13]. However, because immunosuppressants, such as tacrolimus, cyclosporine, everolimus, and sirolimus, have narrow therapeutic windows, therapeutic drug monitoring and dose adjustments remain essential [6, 15]. Importantly, in the event of virological failure, resistance to CAB + RPV LA may limit rescue options. Protease inhibitor-based regimens, often used as salvage therapy, are commonly boosted with cobicistat, which poses a high interaction risk with calcineurin and mTOR inhibitors [16].

In our study, immunosuppressant levels were monitored monthly or bimonthly. Despite minor fluctuations, no patient experienced sustained deviations or loss of control. While the follow-up duration differed pre- and post-switch (15 vs 42 months), which may explain greater variability, no dose changes were required, and management remained stable. Two patients with baseline renal dysfunction (due to CKD or transplant-related disease) tolerated the regimen without incident, which is relevant when interpreting plasma levels.

Although our results support the use of CAB + RPV LA in stable transplant recipients, the posttransplant delay in treatment initiation (1–12 years) limits their applicability to the early peritransplant period. We did not systematically assess rejection, and its absence can only be inferred indirectly. Further studies should evaluate the safety of LA-ART in the immediate posttransplant setting.

CAB + RPV LA's favorable tolerability supports its potential role in transplant patients. With routine monitoring already required in this population, the reduced pill burden of LA-ART may enhance adherence. Current guidelines recommend ART modification pretransplant only if strictly necessary and well in advance [6, 9]; thus, switching solely to reduce pills is not justified. However, in cases of ART incompatibility with future immunosuppressants, CAB + RPV LA may be considered based on these findings.

One patient had nontransplant-related renal insufficiency (baseline GFR 32.07 mL/min), and another met CKD criteria (GFR 23.83 mL/min) with cirrhosis. All patients received nephrotoxic or hepatotoxic agents alongside ART. Current data support CAB + RPV LA use in renal impairment [17], including in hemodialysis [11]. No differences have been found in patients with mild/moderate hepatic insufficiency, though data in Child–Pugh class C are lacking.

Renal and liver function were routinely monitored. Liver enzymes (AST, ALT, bilirubin, AP, and GGT) remained within expected ranges and aligned with historical values. Our data support the safety of CAB + RPV LA in patients with renal or hepatic dysfunction, including transplant recipients. It is also important to note that CAB + RPV LA is contraindicated in patients with active HBV infection. Among our cohort, 3 unvaccinated individuals showed serologic profiles consistent with past HBV exposure without active infection. No HBV-related events occurred, but this remains an important clinical consideration in clinical decision-making.

Injection-related complications are a theoretical concern [11, 18], especially in patients with thrombocytopenia or on anticoagulation [5]. One patient, who started CAB + RPV LA on 9 February 2024, was on Apixaban 5 mg every 12 hours for bilateral deep vein thrombosis due to a prothrombotic oncological state. Anticoagulation was discontinued on 16 April 2024, after Doppler-confirmed resolution. During this period, 2 CAB + RPV LA doses were administered without complications, and no bridging with oral CAB or RPV was needed, highlighting the importance of individualized management. None of the 5 patients, including the one on full-dose Apixaban, reported pain, bruising, bleeding at the injection sites, or any injection-related adverse reactions described in the literature [19–21].

When evaluating the potential benefits of this treatment, the personal impact on the patient must also be considered [18]. Although the switch was proposed by the patient in only 1 of the 5 individuals, all of them have expressed a high level of satisfaction when routinely asked. Bimonthly administration may improve adherence and continuity of care, particularly during travel, hospitalizations, or impaired oral intake.

In summary, CAB + RPV LA appears to be a safe and effective ART strategy in virologically suppressed transplant recipients. It minimizes pill burden, shows no significant pharmacokinetic interference with immunosuppressants, and may improve quality of life in this complex population.

## References

[1] Lu CH, Bednarczyk EM, Catanzaro LM, Shon A, Xu JC, Ma Q. Pharmacokinetic drug interactions of integrase strand transfer inhibitors. Curr Res Pharmacol Drug Discov 2021; 2:100044. 34909672 10.1016/j.crphar.2021.100044 PMC8663927

[2] Harbell J, Terrault NA, Stock P. Solid organ transplants in HIV-infected patients. Curr HIV/AIDS Rep 2013; 10:217–25. 23893004 10.1007/s11904-013-0170-z PMC5899895

[3] Weber ML, Ibrahim HN, Lake JR. Renal dysfunction in liver transplant recipients: evaluation of the critical issues. Liver Transpl 2012; 18:1290–301. 22847917 10.1002/lt.23522

[4] Zheng X, Gong L, Xue W, et al Kidney transplant outcomes in HIV-positive patients: a systematic review and meta-analysis. AIDS Res Ther 2019; 16:37. 31747972 10.1186/s12981-019-0253-z PMC6868853

[5] Srinivas TR, Meier-Kriesche HU, Kaplan B. Pharmacokinetic principles of immunosuppressive drugs. Am J Transplant 2005; 5:207–17. 15643980 10.1111/j.1600-6143.2005.00748.x

[6] U.S. Department of Health and Human Services Panel on Antiretroviral Guidelines for Adults and Adolescents . Transplantation in people with HIV. Clinicalinfo.HIV.gov. 2024 Sep 12 [cited 2025 Jan 25]. Available at: https://clinicalinfo.hiv.gov/en/guidelines/hiv-clinical-guidelines-adult-and-adolescent-arv/transplantation.

[7] Lagoutte-Renosi J, Flammang M, Ducloux D, et al Bictegravir/emtricitabine/tenofovir alafenamide combination in the management of kidney transplant patients with HIV receiving immunosuppressants. J Chemother 2022; 34:199–202. 34180378 10.1080/1120009X.2021.1940436

[8] National Institutes of Health (NIH) . FDA-approved HIV medicines [Internet]. HIV.gov; 2021 [cited 2025 Feb 16]. Available at: https://hivinfo.nih.gov/understanding-hiv/fact-sheets/fda-approved-hiv-medicines.

[9] Blumberg EA, Rogers CC; American Society of Transplantation Infectious Diseases Community of Practice. Solid organ transplantation in the HIV-infected patient: guidelines from the American Society of Transplantation Infectious Diseases Community of Practice. Clin Transplant 2019; 33:e13499. 30773688 10.1111/ctr.13499

[10] Gaur AH, Capparelli EV, Calabrese K, et al Safety and pharmacokinetics of oral and long-acting injectable cabotegravir or long-acting injectable rilpivirine in virologically suppressed adolescents with HIV (IMPAACT 2017/MOCHA): a phase 1/2, multicentre, open-label, non-comparative, dose-finding study. Lancet HIV 2024; 11:e211–21. 38538160 10.1016/S2352-3018(23)00300-4 PMC11213970

[11] Rezzonico LF, Baldassari L, Peracchi F, Merli M, Puoti M, Rossotti R. Use of long-acting cabotegravir + rilpivirine during hemodialysis and solid organ transplantation. AIDS 2023; 37:1491–3. 37395256 10.1097/QAD.0000000000003567

[12] Drug Interaction Checker . University of Liverpool HIV Drug Interactions [Internet]. 2025 [cited 2025 Jan 25]. Available at: https://www.hiv-druginteractions.org/.

[13] Coppinger C, Anderson PL. Considerations for drug–drug interactions between long-acting antiretrovirals and immunosuppressants for solid organ transplantation. Expert Opin Drug Metab Toxicol 2025; 21:343–6. 39757464 10.1080/17425255.2024.2448970

[14] Sinxadi PZ, Khoo SH, Boffito M. Pharmacokinetic interactions of modern antiretroviral therapy. AIDS 2021; 35(Suppl 2):S145–51. 34848581 10.1097/QAD.0000000000002950

[15] Christians U, Vinks AA, Langman LJ, et al Impact of laboratory practices on interlaboratory variability in therapeutic drug monitoring of immunosuppressive drugs. Ther Drug Monit 2015; 37:718–24. 26291980 10.1097/FTD.0000000000000205

[16] Diaz NA, Ambrosioni J, Tuset M, et al Tacrolimus, sirolimus and everolimus doses in HIV-infected solid-organ recipients, requiring a cobicistat-based antiretroviral regimen: report of three cases and review. Infect Dis Ther 2021; 10:105. 10.1007/s40121-021-00430-w PMC8027707 33830489

[17] Smith GH, Henry WK, Podzamczer D, et al Efficacy, safety, and durability of long-acting cabotegravir and rilpivirine in adults with human immunodeficiency virus type 1 infection: 5-year results from the LATTE-2 study. Open Forum Infect Dis 2021; 8:ofab439. 34557563 10.1093/ofid/ofab439 PMC8454521

[18] Heyer A, Ogunbanjo GA. Adherence to HIV antiretroviral therapy: part I: a review of factors that influence adherence: open forum. S Afr Fam Pract (2004) 2006; 48:5–9.

[19] Fernandez C, van Halsema CL. Evaluating cabotegravir/rilpivirine long-acting, injectable in the treatment of HIV infection: emerging data and therapeutic potential. HIV AIDS (Auckl) 2019; 11:179–92. 31447590 10.2147/HIV.S184642 PMC6682757

[20] Teichner P, Chamay N, Elliot E, et al Cabotegravir+ rilpivirine long-acting: overview of injection guidance, injection site reactions, and best practices for intramuscular injection administration. Open Forum Infect Dis 2024; 11:ofae282. 38882931 10.1093/ofid/ofae282 PMC11179104

[21] Wang W, Zhao S, Wu Y, et al Safety and efficacy of long-acting injectable agents for HIV-1: systematic review and meta-analysis. JMIR Public Health Surveill 2023; 9:e46767. 37498645 10.2196/46767 PMC10415942

